# Changes in Motor Skill Performance of 13-Year-Old Japanese Boys and Girls: A Cross-Sectional Study over Six Decades (1964–2023)

**DOI:** 10.3390/sports13060173

**Published:** 2025-05-31

**Authors:** Yukitomo Yogi

**Affiliations:** Department of Sports Sociology and Health Sciences, Faculty of Sociology, Kyoto Sangyo University, Kyoto 603-8555, Japan; yogi@cc.kyoto-su.ac.jp

**Keywords:** motor skills, physical fitness, adolescent, physical education, environmental factors, body size, COVID-19 pandemic

## Abstract

This study examines six decades (1964–2023) of changes in motor skills and body dimensions among Japanese 13-year-old students, analyzing grip strength, handball throwing, 50 m dash, endurance running, and composite scores. National Physical Fitness and Motor Skills Survey data were analyzed alongside School Health Examination Survey measurements to identify trends and correlations between performance metrics and anthropometric variables. The results revealed distinct developmental patterns, with motor skills peaking in the 1980s for both genders, followed by decline until 2000, after which boys experienced stagnation while girls showed improvement until 2019. Both genders demonstrated marked decreases following 2020, coinciding with the COVID-19 pandemic. Notably, while height increased significantly over the study period, only boys’ 50 m dash performance showed strong positive correlations with height (r = 0.779) and BMI (r = 0.854). This longitudinal analysis demonstrates how interdisciplinary factors—including educational policy shifts, reduced physical education curriculum hours, changes in urban park design, diminished outdoor play opportunities, and increased sedentary behaviors—collectively impact children’s motor development. These findings hold significant implications for public health initiatives and sports education strategies aimed at reversing concerning trends in youth physical capabilities and addressing the substantial post-pandemic decline in motor performance.

## 1. Introduction

The state of children’s declining physical fitness has been reported in many studies worldwide [1,2,3,4,5,6]. The decline in children’s physical fitness is widely recognized in Japan [7,8,9,10]. Physical fitness supports human development and growth and is necessary for engaging in creative activities as human beings. As Japan faces a rapidly aging demographic structure, with one of the world’s highest proportions of elderly citizens, there are concerns about the declining physical fitness of children, who will lead the country in the future [11]. In Japan, a national government-led annual survey of physical fitness and motor skills has been conducted since 1964 to clarify the status of the nation’s physical fitness and motor skills and to obtain basic data for the guidance and administration of physical education and sports [12]. The results of this survey are used to evaluate the actual physical fitness of children. The Physical Fitness and Motor Skills Survey was conducted from 1964 to 1998 under the name of the Sports Test. However, in 1999, the test was renamed the New Physical Fitness Test after a review of the changes in the nation’s physique and the appropriateness of the test items. The survey comprised eight test items: four basic motor skills (grip strength, handball throwing, 50 m dash, and endurance running), which have been conducted continuously since the beginning of the survey in 1964, and four tests newly introduced in 1998 (crunches, seated forward bend, side-stepping, and standing long jump). The results of the survey are published on the Sports Agency’s website and on the government statistics portal site [13,14].

Prior studies have examined changes over time among Japanese children using data from the Survey of Physical Fitness and Motor Skills. For example, Nishijima et al. (2003) used data from 1964 to 1997 to determine changes over time in physical fitness and motor skills among children aged between 12 and 17 years old [15]. Noi et al. (2002) analyzed data from 1965 to 1998 to determine changes over time for 11-, 14-, and 17-year-old children [16]. Yogi et al. (2014) assessed data from 1964 to 2010 to identify changes over time for 13-year-old children [17]. These studies have indicated that the social and living environments surrounding children influence how this group changes over time [16,17]. Assuming that changes in the environment surrounding children affect their physical fitness, it is necessary to examine the trends of change since 2011.

The Sports Agency reported a gradual improvement in total physical fitness scores for middle school students between 2010 and 2019 [18]. The negative impact of the COVID-19 outbreak, which occurred in December 2019, on children’s fitness has been reported in many international journals [19,20,21,22,23,24,25]. Inevitably, the COVID-19 pandemic has drastically changed the social and living environment through nationwide school closures, restrictions on outdoor activities, reduced physical activity, and increased screen time [26]. Based on 58 years of data from 1964 to 2021 for elementary school students (aged 11 years), Yogi et al. (2024) determined the changes over time in four categories of motor skills (grip strength, softball throwing, 50 m dash, and side-stepping) and the study identified the potential impact of the goals and number of hours allocated to physical education in the Courses of Study [27]. Takahashi et al. (2010) highlights that the Courses of Study for that period shifted the direction of goals from an emphasis on physical fitness to an emphasis on fun [28]. However, the impact of COVID-19 has not been considered in depth. In addition, given that the Courses of Study differ between elementary and junior high schools, it is necessary to examine the background factors that influence changes in junior high school students’ physical fitness over time. Furthermore, only the two tests of grip strength and 50 m dash are the same for elementary and junior high schools; other tests designated for assessing basic motor skills are different. Therefore, it is necessary to identify changes over time among junior high school students.

Previous studies have reported that children’s physical fitness is declining, although it is improving with respect to physique [29,30,31]. Fujii et al. (2006) noted that many reports indicate a decline in motor skills despite the improvement in physique [30]. Yamauchi (2017) also acknowledges the improvement in physique and highlights that grip strength and throwing ability have declined [31]. In Japan, the School Health Examination Survey has been conducted annually since 1900 for clarifying the state of development and health of students in schools [32]. Growth status measurements include height and weight, for which more than 120 years of data exist. Therefore, it would be necessary to examine the effects of changes in body size on motor skills since 1964, when the Physical Fitness and Motor Skills Survey was initiated.

In this study, data spanning 60 years from 1964 to 2023 were used to determine changes over time in the motor skills and body size of Japanese junior high school students (aged 13 years). The study aimed to obtain findings that can be used to improve future physical education and sports coaching. To this end, it assessed the relationship between motor skills and physique and determined the potential background factors influencing the periods of improvement and decline in motor skills from various perspectives, including social and living environments.

## 2. Materials and Methods

### 2.1. Physical Fitness and Motor Skills Survey

The study covered four motor skills (grip strength, handball throwing, 50 m dash, and endurance running) from 1964 to 2023, as published by the Sports Agency (and before 2015 by the Ministry of Education, Culture, Sports, Science and Technology).

Grip strength is measured twice, alternating left and right hands, using a Smedley grip strength meter, and the best result is averaged and rounded to the nearest kilogram. The 50 m dash is measured only once from a crouching start, with times recorded in increments of 1/10th of a second, rounded up to the nearest 1/10th of a second. Endurance is measured only once in the 1500 m run for boys and 1000 m run for girls, with times recorded in seconds. In handball throwing, a size 2 ball (circumference 54–56 cm, weight 325–400 g) is used, which is thrown twice with the dominant hand, with the better record being recorded. Distances are recorded in meters. Detailed measurement methods are clearly defined in the guidelines set forth by the Sports Agency [33].

The data (sample size, mean, and standard deviation) that have been accumulated over 60 years since 1964 target only 13-year-old children. This age is particularly relevant as it follows what research identifies as the “golden age” (9–12 years) of physical development, when the nervous system development is complete and habitual exercise established during childhood often continues into adulthood [34]. Additionally, 13-year-olds are in their second year of junior high school in Japan, when most students have adapted to the educational environment change from elementary school, providing more consistent measurement conditions. A random sampling method was used to select the participants, and care was taken to avoid regional bias. The survey data are guaranteed to be representative of the population, as the survey was conducted by the government. The sample size varies from year to year but generally ranges from 1000 to 3000.

### 2.2. School Health Examination Survey

Data of the School Health Examination Survey published by the Ministry of Education, Culture, Sports, Science and Technology were also used [32]. This survey has been conducted annually since 1900. This study used height and weight data for 13-year-old children from 1964, when the Physical Fitness and Motor Skills Survey began, to 2023. A random sampling method was employed to recruit participants [35]. The survey is also conducted under the auspices of the national government, thus ensuring a good level of representation in the results.

### 2.3. Analysis

To acquire a comprehensive picture of the overall changes over time in the average scores for the four tests (grip strength, handball throwing, 50 m dash, and endurance running), this study presents the records for the 60-year period from 1964 to 2023, standardized by testing (Z-score) and presented in a graph by gender. Standardization was performed using the following equation.

$$z=\frac{X-\mu }{\sigma }$$

(X: Recorded values for each year and each category from 1964 to 2023; μ: Mean value for the period; σ: Standard deviation for the period).

Next, in order to graph the change over time by test based on the recorded values, the starting points for periods of improvement and decline were established by examining changes over time by test in this study. To determine the starting point of periods of improvement and decline, the average values in the years before and after the highest years during the improvement period and the lowest years during the decline period were calculated, referring to previous studies that examined long-term trends in physical fitness [15]. The mean values calculated in this manner were then used to examine whether there was a significant difference between the periods of improvement and decline. The starting point for periods of improvement and decline varied for each motor skill test. When there were two starting points, a *t*-test was used. Some motor skill tests with repeated periods of improvement and decline have three or more starting points. In such cases, a one-way analysis of variance was used. When significant differences were found, multiple comparison tests were performed. Standardized Z-values were used to examine significant differences. In this study, the coefficient of determination was determined for each graph, and the R^2^ value indicated the extent to which the objective variable can be explained by the explanatory variables. The R^2^ value was calculated using the following equation.

$$R^{2}=1-\frac{\sum_{i=1}^{n}{\left(y_{i}-{\hat{y}}_{i}\right)}^{2}}{\sum_{i=1}^{n}{\left(y_{i}-\bar{y}\right)}^{2}}$$

(n: Number of data, $y_{i}$: Observed values, ${\hat{y}}_{i}$: Predicted values, $\bar{y}$: Mean of observed values)

Height and weight data from the School Health Examination Survey from 1964 to 2023 were used to examine changes over time. In addition, body mass index (BMI) was calculated based on height and weight, and the relationship between the four tests and body size was examined using Pearson’s correlation analysis. Correlation strength was defined as r0.7–0.9 = very strongly correlated, r0.4–0.7 = somewhat correlated, r0.2–0.4 = weakly correlated, and r0–0.2 = almost uncorrelated.

Excel 2016 and SPSS Statistics 28 were used for statistical processing in this study. The statistical significance level was set at *p* < 0.05.

## 3. Results

### 3.1. Changes over Time in Motor Skills (Average Scores of Four Tests)

**Figure 1:**
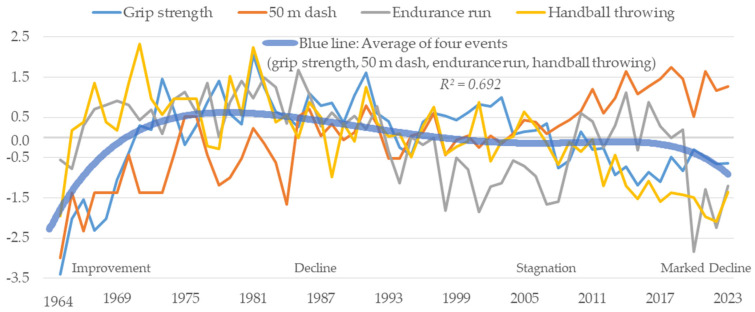
Changes over time in motor skills for boys.

Figure 1 shows the changes over time for boys. There was a notable improvement in the changes over time in the average scores of the four tests from 1964 to the 1980s. Scores were highest around 1985–1987. After this peak, there was a decline till roughly the year 2000. From 2000 to 2019, stagnation was observed. However, a marked downward trend was seen again after 2020. There was a clear period of improvement, a period of decline, a period of stagnation, and a period of marked decline in the changes over time in the average scores of the four tests for boys. Characteristically, from the 1960s to around 2000, the four tests followed somewhat similar trends. However, since 2000, only the 50 m dash showed an improving trend, which is different from the other three events.

**Figure 2:**
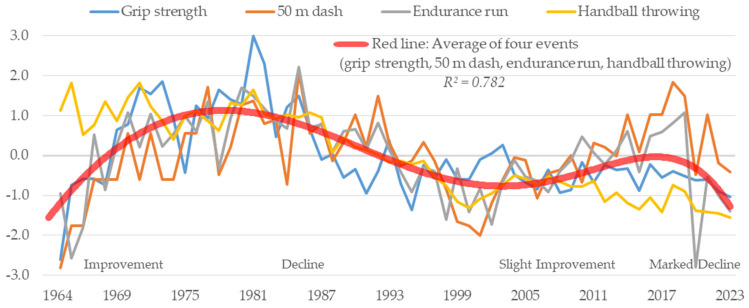
Changes over time in motor skills for girls.

Figure 2 shows the changes over time for girls. Looking at the changes over time in the average scores of the four tests, there is a noticeable improvement from 1964 to the 1980s. Scores were highest around 1980–1982. After this peak, there was a decline till roughly the year 2000. The trend of gradual improvement continued from 2000 to 2019. However, as with boys, there was a marked downward trend after 2020. There was a clear period of improvement, a period of decline, a period of slight improvement, and a period of marked decline in the changes over time in the average scores of the four tests for girls. In contrast to boys, the overall changes in the four tests were characterized by somewhat similar trends. In addition, girls did not experience a period of stagnation; there have only been improving or declining phases for girls.

### 3.2. Changes over Time by Category

**Figure 3:**
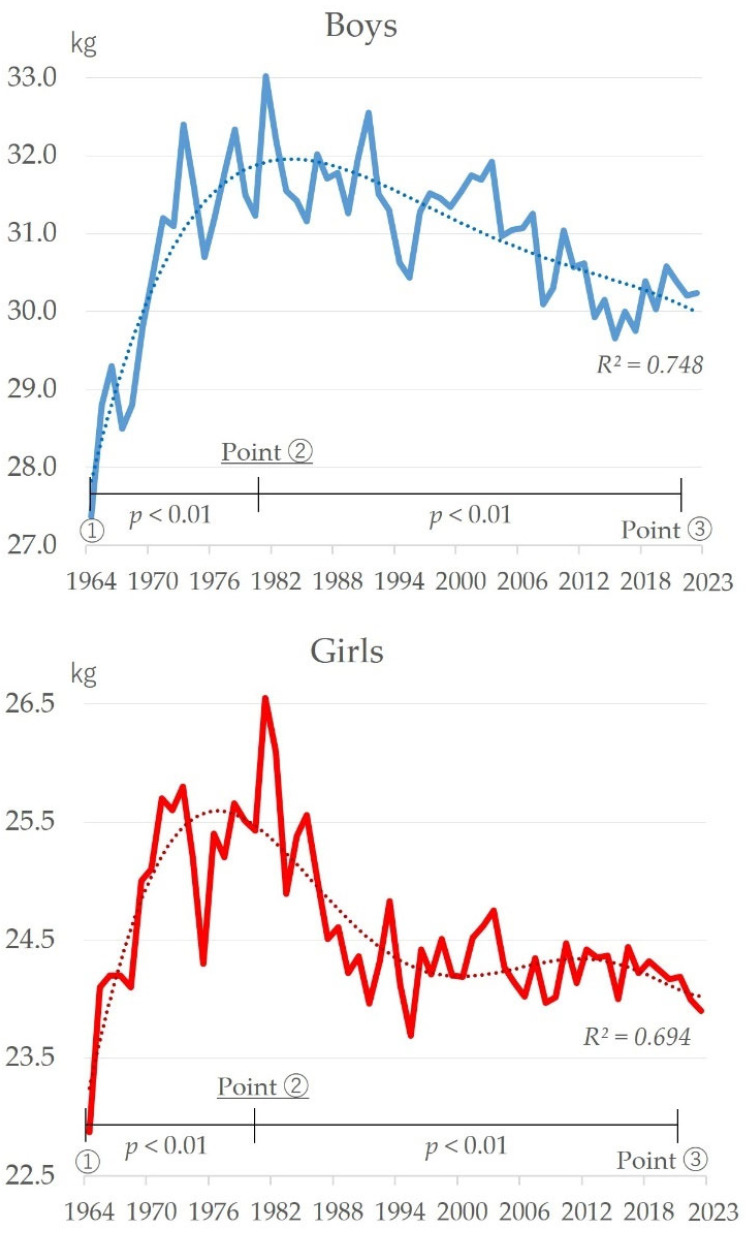
Changes over time in grip strength for boys and girls.

Figure 3 shows the changes in grip strength over time. There was an improvement for both boys and girls from 1964 to about 1980, but there has since been a decline (boys: *R*^2^ = 0.748; girls: *R*^2^ = 0.694). The highest recorded values for both boys and girls were in 1981 (33.02 kg for boys and 26.55 kg for girls), and the lowest since then were in 2015 (29.66 kg) for boys and 1995 (23.69 kg) for girls. Therefore, significant changes between the periods of improvement and decline were seen based on a comparison of the three starting points at around 1964 when the survey began, around 1981 when peak values were recorded, and around 2022 when values were in decline (boys: F = 16.37, *p* < 0.01; girls: F = 15.86, *p* < 0.01). Multiple comparison tests showed that the rates around 1981 (Point ②) were significantly higher than those around Points ① and ③ for both boys and girls (multiple comparison tests: Point ② > Point ①, ③).

**Figure 4:**
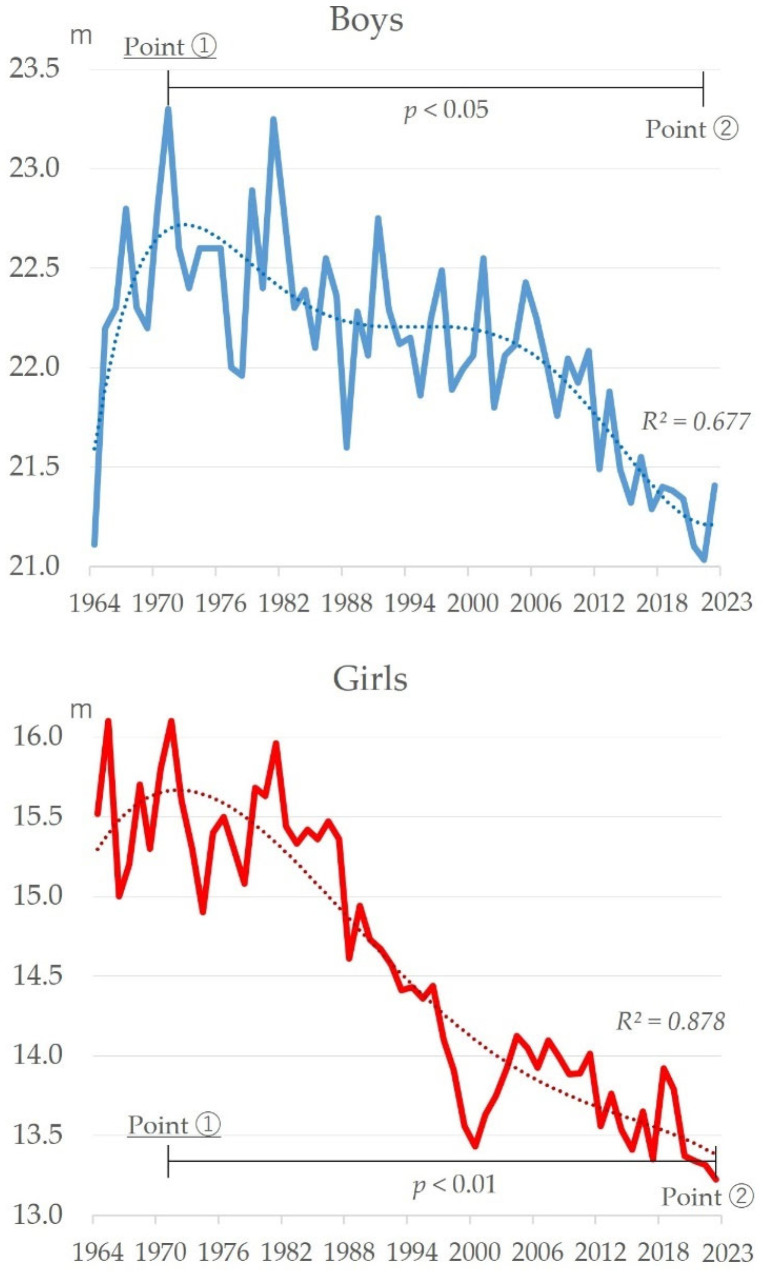
Changes over time in handball throwing for boys and girls.

Figure 4 shows the changes over time for handball throwing. Both boys and girls showed an improving trend from about 1964 to 1970 and a declining trend since then (boys: *R*^2^ = 0.677, girls: *R*^2^ = 0.878). The highest recorded values for both boys and girls were in 1971 (23.30 m for boys and 16.10 m for girls; the same values as those in 1965), and the lowest since then were in 2022 (21.03 m) for boys and 2023 (13.22 m) for girls. When comparing the highest point at around 1971 and the lowest point since then (around 2022 for boys and 2023 for girls), there was a significant decrease (boys: t = 7.55, *p* < 0.05; girls: t = 17.65, *p* < 0.01).

**Figure 5:**
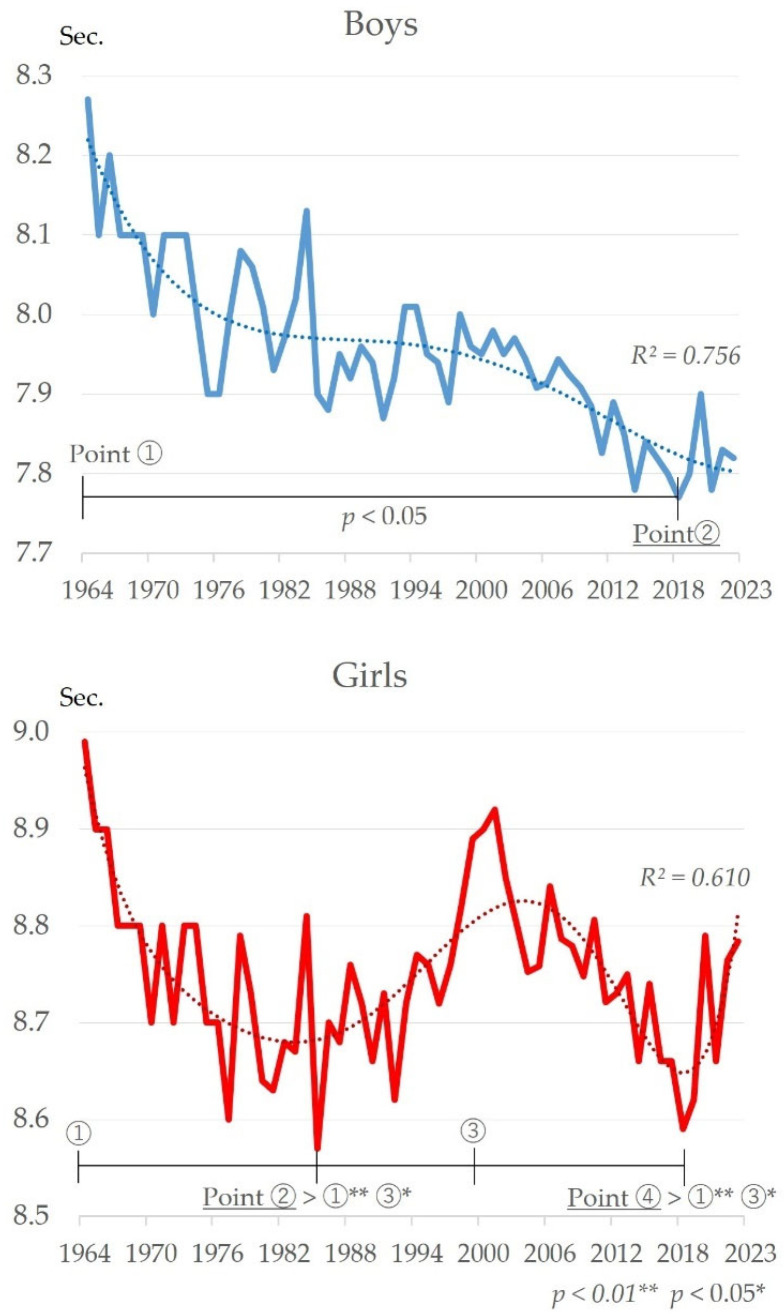
Changes over time in 50 m dash for boys and girls.

Figure 5 shows the changes over time in the 50 m dash. Boys show a long-term improving trend from 1964 to 2023 (*R*^2^ = 0.756). The highest recorded value was in 1964 (8.27 s) and the lowest in 2018 (7.77 s). Comparing the highest point around 1964 to the lowest point around 2018, a significant improvement was found (t = −6.93, *p* < 0.05). Girls tended to improve from about 1964 to 1985, declined from about 1986 to 2000, improved from then to about 2018, and declined again after 2020 (*R*^2^ = 0.610). Therefore, comparing each starting point, there was a significant change between the improvement and decline periods (F = 11.57, *p* < 0.01). The results of multiple comparison tests showed significant lows around 1985 (Point ②) and around 2018 (Point ④) (multiple comparison tests: Point ②, ④ > ①, ③).

**Figure 6:**
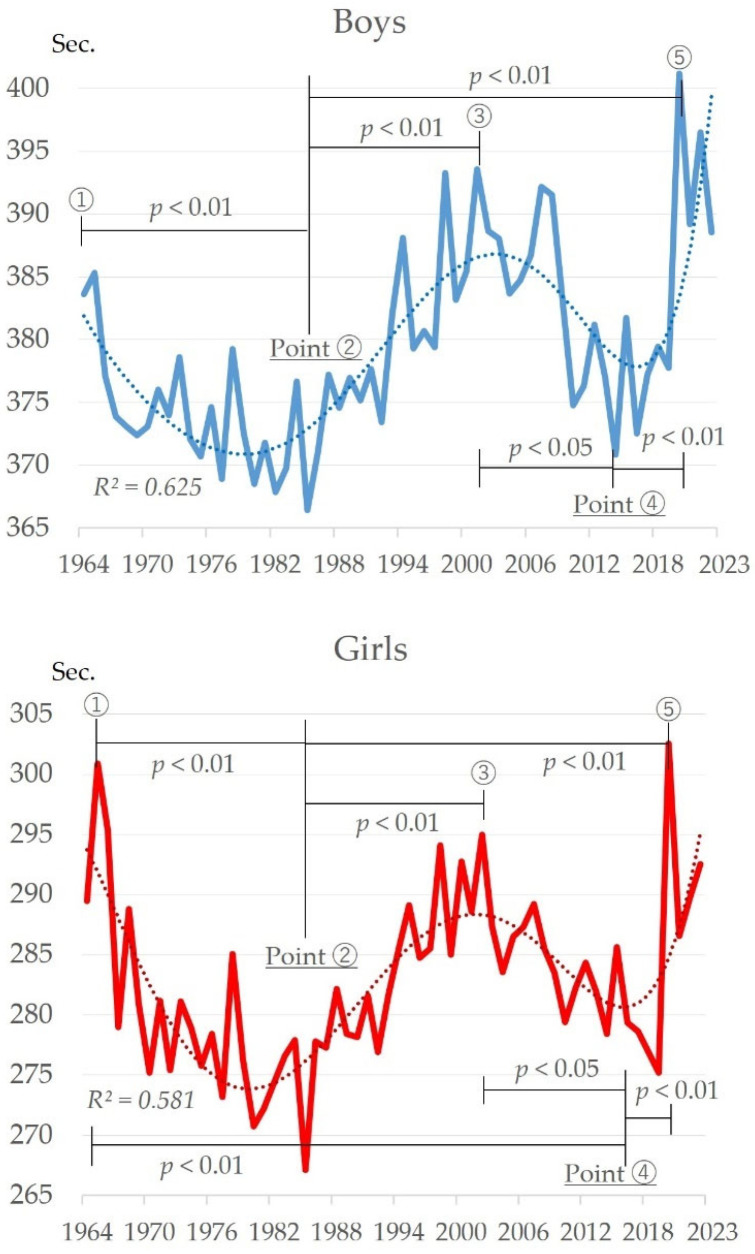
Changes over time in endurance run for boys and girls.

Figure 6 shows the changes over time in the endurance run (1500 m run (boys) and 1000 m run (girls)). Both boys and girls tended to improve from 1964 to about 1985, declined from about 1985 to about 2000, improved again from about 2000 to about 2017, and have declined since then (boys: *R*^2^ = 0.625; girls: *R*^2^ = 0.581). The highest recorded values for both boys and girls were in 2020 (401.17 s for boys and 302.54 s for girls) and the lowest in 1985 (366.40 s for boys and 267.11 s for girls). One characteristic of endurance running is that both boys and girls go through periods of improvement and decline. Therefore, comparing each starting point, there was a significant change between the improving and declining periods (boys: F = 16.75, *p* < 0.01; girls: F = 20.86, *p* < 0.01). Multiple comparison tests showed significant low points for boys around 1985 (Point ②) and 2017 (Point ④). Significant highs were also observed around 2020 (Point ⑤) (multiple comparison tests: Point ② > Point ①, ③, ⑤, Point ④ > Point ③, ⑤). The results for girls were similar to those for boys (multiple comparison tests: Point ②, ④ > Point ①, ③, ⑤).

### 3.3. Changes in Physique

**Figure 7:**
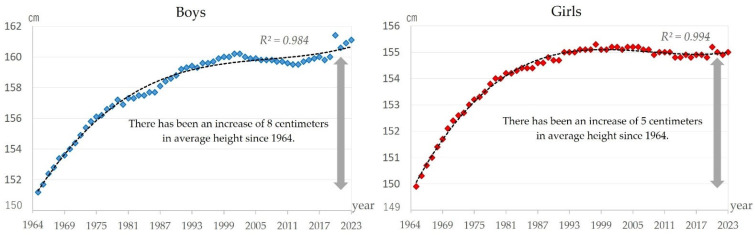
Changes over time in height for boys and girls.

Figure 7 shows the changes in height over time. Height increased markedly between 1964 and about 2000 by about 8 cm for boys and 5 cm for girls. However, since 2000, the trend has been stagnant (boys: *R*^2^ = 0.984; girls: *R*^2^ = 0.994).

### 3.4. Relationship Between Motor Skills and Body Size

Table 1 shows the results of the correlation analysis between motor skills and height, weight, and BMI. There was a very strong positive correlation among boys between the 50 m dash and height (r = 0.779, *p* < 0.01), weight (r = 0.826, *p* < 0.01), and BMI (r = 0.854, *p* < 0.01). No strong correlation was observed among girls.

**Figure 8:**
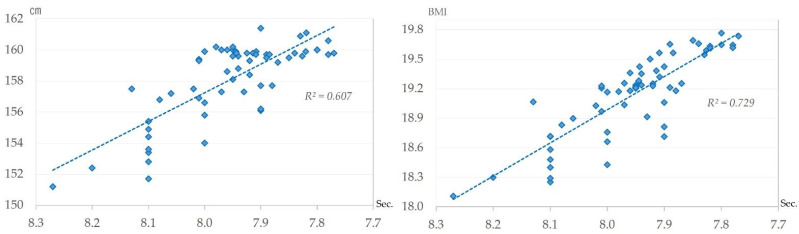
Relationship between 50 m dash result and height and 50 m dash result and BMI for boys.

Figure 8 shows the relationship between the 50 m dash result and height and 50 m dash result and BMI for boys. This figure explains how height (*R*^2^ = 0.607) and BMI (*R*^2^ = 0.729) are positively correlated with improvement in the 50 m dash record.

## 4. Discussion

In this study, the author determined changes in motor skills and body size over time (Figure 1, Figure 2, Figure 3, Figure 4, Figure 5, Figure 6 and Figure 7). The relationship between motor skills and body size was also presented (Table 1, Figure 8). In the Section 4, the author will examine the background factors influencing the results obtained in this study from the viewpoints of social and living environment, including changes in the average scores of the four tests over time, changes over time by test, and the relationship between tests and body size.

### 4.1. Changes over Time in the Average Scores of Four Tests

This study revealed changes over time in the motor skills of boys and girls (Figure 1 and Figure 2). The average scores of the four tests improved from 1964 to the 1980s for both boys and girls. From the late 1980s to around 2000, there was a decline for both boys and girls. Yogi et al. (2014) stated that the improvement from 1964 to the 1980s was influenced by the goals of physical fitness in the Courses of Study at that time [17]. The Courses of Study are revised approximately every 10 years in response to the demands of society. In the 1969 Courses of Study, physical education reached a peak in hours of instruction at 125 h per year [36]. It has also been reported that Japan had been making national efforts to improve the physical fitness of its citizens since before 1964 [37,38]. This social environment may have influenced the development of motor skills.

The decline from the late 1980s to around 2000 may also be attributable to the Courses of Study. In the 1977 and 1989 Courses of Study, the number of hours of physical education was reduced to 105 h per year [39,40]. Therefore, it can be inferred that the reduction in the number of class hours due to the shift in goals and the accompanying changes in the curriculum led to a decrease in physical activity in physical education, which in turn affected the decline in motor skills. In addition, the growing tendency in the public consciousness to prioritize academic achievement over outdoor play and sports has led to a polarized view of physical activity. This change in the environment may be another factor.

From 2000 to 2019, values for boys stagnated, while those for girls gradually improved. The 1998 Courses of Study introduced a new curriculum to improve physical fitness in elementary and junior high schools in response to the long-term decline that had been ongoing since the late 1980s [41]. It is believed that this response halted the decline. Why, then, was a gradual improvement seen among girls? In Japan, athletic club activities such as basketball and volleyball are offered in almost all junior high schools. The participation rate in athletic club activities during this period was around 78% for boys and 57% for girls, showing a marked gender difference [42]. Gender differences in participation rates consequently affect physical fitness and motor skills. It has been reported that the difference in physical activity between boys and girls widens with age during youth [43,44]. In addition, girls tend to be less physically active than boys in relation to biological maturation, highlighting the need to increase physical activity among girls [43,45]. Therefore, the legally binding Courses of Study introduced a new curriculum aimed at improving physical fitness, providing girls who did not participate in athletic club activities with an opportunity to engage in physical activity through physical education. It can be inferred that the increase in physical activity through physical education led to a gradual improvement in the overall motor skills of girls who did not participate in athletic club activities.

After 2020, there has been a marked decline in motor skills for both boys and girls. This may be attributable to the fact that the COVID-19 pandemic drastically changed the social and living environment. In Japan, as in other countries, students were asked to stay at home and schools were closed for approximately 3 months nationwide from March to May 2020, with additional regional closures extending into 2021 [46].As a result, children were unable to engage in physical education and sports and opportunities for physical activity were lost. Schools resumed classes online, but there were limitations in physical education. This is probably the reason for the marked decline after 2020.

### 4.2. Changes in Grip Strength over Time

Grip strength has shown a long-term decline since the mid-1980s for both boys and girls (Figure 3). It has been highlighted that grip strength is impacted by lifestyle factors [47]. Naito (2013) has shown that the evolution of water faucet handles has reduced individuals’ application of the gripping action, which is related to the decline in grip strength [48]. In addition, in children’s living environments, there have been progressive changes in the design of bags and doors. Children commonly used to carry bags that contained all of their necessary belongings. These bags were held alternately with the right and left hands to deal with fatigue. This gripping action would be applied unconsciously. Today, however, backpacks are carried on the back and both hands are free. Many children can be seen using smartphones with their free hands. Furthermore, the gripping action that used to occur unconsciously when opening and closing doors has now become unnecessary due to the increasing prevalence of automatic doors. In terms of the social environment, changes in park maintenance should be taken into account. As Japan’s population ages, health-related playground equipment and walking trails are being installed for older adults. As a result, pieces of playground equipment intended for children to support their own weight, such as iron bars and climbing frames, have decreased in number. In addition, while the Courses of Study explicitly include skills using bar exercises, schools can choose between a vaulting exercise, mat exercise, and balance beam exercise. In reality, few schools actually choose the iron bar exercise. These actual conditions and changes in the environment have led to a decrease in gripping movements, and the accumulation of such movements is a potential background factor influencing the decline in grip strength.

### 4.3. Changes in Handball Throwing over Time

Handball throwing, like grip strength, has shown a long-term decline since the mid-1980s for both boys and girls (Figure 4). This study found that height and stature have continued to increase since the 1980s (Figure 7). Generally speaking, a taller stature is considered to give the thrower a higher release point when throwing the ball, which is advantageous to the thrower. Why, then, is there a downward trend? In terms of the living environment, a decrease in children’s outdoor play has been reported [49]. In the 1970s, sports facilities were not well developed in Japan [50]. Therefore, outdoor play in public squares and alleys was dominated by ball games [51]. However, from that time on, the construction of sports facilities increased [52]. This has given children the opportunity to experience many sports, including indoor sports such as badminton and table tennis and outdoor sports such as soccer and tennis. In terms of the social environment, the banning of ball games from public parks may have had an impact. An increasing number of parks are banning baseball and other ball games for the safety of park visitors and due to the issue of balls entering nearby private property. Currently, 60% of urban parks prohibit ball games [53]. This situation and changes in the environment may have led to a decrease in throwing movements, and the combination of these background factors have influenced the decline in handball throwing.

### 4.4. Changes in 50 m Dash over Time

The change trend of changes in the 50 m dash is different for boys and girls (Figure 5). Boys show a long-term improving trend. Yogi et al. (2014) identified that the improvement in the 50 m dash records since 1977 are attributed to increased soccer participation, as soccer develops instantaneous force and speed, with performance further enhanced by 1993 launch of the professional league [17]. Changes in living conditions may be a contributing factor, as the establishment of professional leagues has led to an increase in the number of soccer clubs in the region and more media coverage of the sport. Furthermore, this study revealed an increase in body size (Figure 7). Correlation analysis showed a very strong positive correlation with body size (height, weight, and BMI) (Table 1). The results indicated that an increase in body size may have contributed to stronger leg strength, while an increase in height may have contributed to an increase in stride length. The evolution of sports shoes should also be taken into consideration. Shoe manufacturers have made significant advances in sport shoe functionality and weight reduction over the past 60 years. In addition, schools have strengthened specific measures to prevent accidents during physical education activities, and a provision on safety management was added to the School Health and Safety Law in 2009 [54]. This has significantly improved the condition of playgrounds. Such changes in the social environment may have influenced the improvement of the record for the 50 m dash, which is measured in 0.1-s increments.

For girls, in contrast, there has been both an improvement and a decline. In terms of changes in the living environment, it was reported that outdoor play decreased and indoor play increased from the late 1980s until around 2000 [55]. The accompanying decrease in physical activity may have contributed to the decline during this period. Regarding the improvement from the late 2000s to 2019, changes in the social environment may have had an impact. The Basic Plan for the Promotion of Sports formulated in 2000 set out to improve the nation’s physical fitness, and efforts were undertaken to improve exercise programs in schools and communities [56]. As mentioned earlier, girls tend to be less physically active than boys in relation to biological maturation, and the improvement is attributed to increased physical activity as a result of government-led measures. Dramatic environmental change brought about by the COVID-19 pandemic since 2020 may have influenced the differing trends for boys and girls, as expressed in their biological maturation.

### 4.5. Changes in Endurance Running over Time

Endurance running has been improving and declining among both boys and girls (Figure 6). The decline from the 1980s to around 2000 can be attributed to the explosion of video games since the 1980s, which drastically changed the living environment. Previous studies have reported that physical activity is positively related to aerobic exercise [57]. Thus, the prevalence of video games may have led to an increase in sedentary behavior, reducing physical activity and affecting endurance. Following a long-term decline since the 1980s, the improvement from mid-2000 to around 2018 may have been influenced by the introduction of a new curriculum aimed at improving physical fitness in the 1998 Courses of Study [41]. For students whose outdoor play has decreased and sedentary behavior has increased, physical education provides a valuable opportunity for physical activity. It is thought that cardiopulmonary function and endurance were improved by physical activities in the physical education program, with the aim of improving physical fitness. In Japan, it has been reported that the percentage of persons with obesity aged 6–14 years increased between 1985 and 2015 [31]. However, the physical education curriculum may have played an important role in the improvement in endurance running from the mid-2000s to 2018.

Since 2020, there has been a marked decline for both boys and girls. This factor is possibly attributable in large part to the change in the environment caused by the COVID-19 pandemic. Endurance running is an important indicator of general endurance as well as cardiorespiratory and muscular endurance [58]. The COVID-19 pandemic markedly reduced physical activity and directly affected endurance running, which measures the body’s ability to move in a sustained manner. Decreased general endurance suggests negative effects on cardiopulmonary and muscular endurance. There are risks of negative health effects in the future, such as an increase in obesity and lifestyle-related diseases.

### 4.6. Physique Changes and Their Limited Impact on Motor Performance

Height increased markedly over the 60 years for both genders (Figure 7). This trend has been attributed to the fact that people have become more wealthy, and they can now eat more nutritious food [59]. Body size is noted to have been affected by nutritional status, and nutritional status is affected by economic status [31]. Thus, it is likely that people’s dietary habits were changed by the enhancement in social life from the high economic growth that lasted for about 20 years from 1955 to 1973, which contributed to the increase in body size. In general, an increase in body size is thought to increase muscle mass, which in turn improves physical fitness. However, compared to their parents’ generation, today’s children have improved in physique but declined in terms of physical fitness [31,59]. The present study has presented a very strong positive correlation between an improvement in 50 m dash records and body size in boys, but a similar relationship was not observed in other tests or among girls. Therefore, the effect of increased body size on motor skills may be limited.

It is important to acknowledge that, over the six decades covered in this study, there have been documented changes in the timing of biological maturation among Japanese adolescents. Earlier maturation could affect physical performance metrics independently of environmental factors. Studies have shown that the age of menarche has decreased in Japan during portions of our study period, and skeletal maturation patterns have also shifted [60,61]. These biological changes could have influenced our data, particularly during periods of significant secular changes in growth patterns. Future research should attempt to control for maturation timing when examining the long-term trends in motor skill performance.

## 5. Conclusions

Using data published by the Sports Agency and the Ministry of Education, Culture, Sports, Science and Technology, this study could determine changes in motor skills and body size and the relationship between motor skills and body size among Japanese junior high school students (aged 13 years). Changes in the environments surrounding the children are thought to influence changes in motor skills over time. In terms of the social environment, changes in the Courses of Study, which are revised every 10 years, and the state of park maintenance due to the aging of society may have had an impact. In the living environment, a decrease in outdoor play, an increase in sedentary behavior, and the evolution of bags and sports shoes may have each had an effect.

The results presented in this study and the examination of potential environmental factors influencing the changes studied herein provide useful insights for improving physical education and sports instruction in the future. However, the impact of changes in the social and living environments on motor skills is based on a comprehensive overview of various phenomena reported in previous studies and reports, not on a variable analysis of these phenomena. It is also important to note the limitation that the instruments used in this study (grip tester, stopwatch, and handballs) have evolved over the past 60 years, which may have had some impact on the results of this study.

## Figures and Tables

**Table 1:** Results of correlation analysis between motor skills and height, weight, and BMI.

Gender						Boys		
Events/Body size	No 1.	No 2.	No 3.	No 4.	No 5.	No 6.	No 7.	No 8.
No 1. Grip strength	**−**	0.143	0.126	0.440 **	0.746 **	0.351 **	0.271 *	0.176
No 2. Handball throwing	0.559 **	**−**	−0.419 **	0.449 **	0.642 **	−0.434 **	−0.496 **	−0.539 **
No 3. 50 m dash	0.349 **	0.089	**−**	−0.116	0.265 *	0.779 **	0.826 **	0.854 **
No 4. Endurance run	0.552 **	0.730 **	0.421 **	**−**	0.637 **	−0.452 **	−0.411 **	−0.314 *
No 5. Four events averages	0.802 **	0.707 **	0.882 **	0.675 **	**−**	0.107	0.083	0.077
No 6. Height	−0.103	0.315 *	0.018	−0.688 **	−0.149	**−**	0.987 **	0.926 **
No 7. Weight	−0.125	0.215	−0.070	−0.694 **	−0.220	0.982 **	**−**	0.975 **
No 8. Body mass index	−0.132	0.069	−0.177	−0.647 **	−0.289*	0.892 **	0.962 **	**−**
Gender			Girls					

*p* < 0.01 **, *p* < 0.05 *.

## Data Availability

Data are available from the corresponding author upon reasonable request.

## References

[1] Filippone B., Vantini C., Bellucci M., Faigenbaum A.D., Casella R., Pesce C. (2007). Trend secolari di involuzione delle capacità motorie in età scolare. SDS.

[2] Radulović A., Jurak G., Leskošek B., Starc G., Blagus R. (2022). Secular trends in physical fitness of Slovenian boys and girls aged 7 to 15 years from 1989 to 2019: A population-based study. Sci. Rep..

[3] Tomkinson G.R., Olds T.S., Kang S.J., Kim D.Y. (2007). Secular trends in the aerobic fitness test performance and body mass index of Korean children and adolescents (1968–2000). Int. J. Sports Med..

[4] Tremblay M.S., Shields M., Laviolette M., Craig C.L., Janssen I., Gorber S.C. (2010). Fitness of Canadian children and youth: Results from the 2007–2009 Canadian Health Measures Survey. Health Rep..

[5] Grant R.T., Luc A.L., Tim S.O., Georges C. (2003). Secular Trends in the Performance of Children and Adolescents (1980–2000): An Analysis of 55 Studies of the 20m Shuttle Run Test in 11 Countries. Sports Med..

[6] Grant R.T., Duncan M., Shingo N., Dae Y.K., Zhengzhen W., Ren H. (2012). Temporal changes in long-distance running performance of Asian children between 1964 and 2009. Sports Med..

[7] Ushijima K., Watanabe H., Shimura M. (2016). The relationships among physical fitness, academic achievements, psychological stress scale scores and lifestyles of junior high school students. Jpn. J. Hum. Growth Dev. Res..

[8] Niimoto S., Yamasaki M. (2013). The relationship between physical strength and physical activity in elementary school children. Jpn. J. Hum. Growth Dev. Res..

[9] Toda S., Watanabe T., Yan T.C. (2007). Relationship between the Amount of Daily Physical Activity, Physical Fitness and Physique of Students in the Upper Grades of Primary School. Jpn. J. School Health.

[10] Kasuga K., Nakano T., Oguri K. (2010). Time of Polarization at the Physical Fitness Level in Children—Based on the Follow-up Survey to the Past of the Group That Exists in Two Poles at Five-Years Old Time-. Jpn. J. Med. Educ..

[11] The Central Council on Education Report, Comprehensive Measures to Improve Children’s Physical Fitness. https://www.mext.go.jp/b_menu/shingi/chukyo/chukyo0/toushin/021001.htm.

[12] Guidelines for Implementation of Physical Fitness and Motor Skills Surveys. https://www.stte-shizuoka.jp/stte/img/r03_chousajissi-youkou_sport.pdf.

[13] Physical Fitness and Motor Skills Survey—Summary of Results. https://www.mext.go.jp/sports/b_menu/toukei/chousa04/tairyoku/kekka/1368159.htm.

[14] e-Stat Japan in the Statistics. https://www.e-stat.go.jp/stat-search/files?page=1&toukei=00402102&tstat=000001088875.

[15] Nishijima T., Kokudo S., Osawa S. (2003). Changes over the years in physical and motor ability in Japanese youth in 1964-97. Int. J. Sport Health Sci..

[16] Noi S., Masaki T. (2002). The educational experiments of school health promotion for the youth in Japan: Analysis of the ‘sport test’ over the past 34 years. Health Promot. Int..

[17] Yogi Y., Kokudo S. (2014). An examination of secular contrast by physical fitness and athletic capabilities of junior high school students. Bull. Grad. Sch. Hum. Dev. Environ. Kobe Univ..

[18] (2020). Physical Fitness and Motor Skills Survey Report. https://www.mext.go.jp/sports/b_menu/toukei/chousa04/tairyoku/kekka/k_detail/1421920_00003.htm.

[19] Dunton G.F., Do B., Wang S.D. (2020). Early effects of the COVID-19 pandemic on physical activity and sedentary behavior in children living in the U.S. BMC Public Health.

[20] Xiang M., Zhang Z., Kuwahara K. (2020). Impact of COVID-19 pandemic on children and adolescents’ lifestyle behavior larger than expected. Prog. Cardiovasc. Dis..

[21] Stefen C.E.S., Bastian A., Alexander B., Ana E., Simon K., Carina N., Claudia N., Doris O., Annette W., Alexander W. (2020). Physical activity and screen time of children and adolescents before and during the COVID-19 lockdown in Germany: A natural experiment. Sci. Rep..

[22] Chen P., Mao L.J., Nassis G.P., Harmer P., Ainsworth B.E., Li F. (2020). Coronavirus disease (COVID-19): The need to maintain regular physical activity while taking precautions. J. Sport Health Sci..

[23] Camille C., Nicole F., Léna P., Pauline G., Alicia F., Audrey B., Line B., Mélina B., Julie S., Terry G. (2021). Adverse Collateral Effects of COVID-19 Public Health Restrictions on Physical Fitness and Cognitive Performance in Primary School Children. Int. J. Environ. Res. Public Health.

[24] Chambonniere C., Lambert C., Fearnbach N., Tardieu M., Fillon A., Genin P., Larras B., Melsens P., Bois J., Pereira B. (2021). Effect of the COVID-19 lockdown on physical activity and sedentary behaviors in French children and adolescents: New results from the ONAPS national survey. Eur. J. Integr. Med..

[25] Wahl-Alexander Z., Camic C.L. (2021). Impact of COVID-19 on School-Aged Male and Female Health-Related Fitness Markers. Pediatr. Exerc. Sci..

[26] Kasai A., Shikano A., Tanaka R., Yoshinaga M., Noi S. (2024). Movement behaviors and subjective health complaints during COVID-19 pandemic related school closure and after school reopening among junior high school students. Nippon Sport Science University. J. Child. Phys. Health.

[27] Yogi Y., Ishikawa Y., Takahashi S. (2024). Secular Contrasts in Physical Fitness and Athletic Skills in Japanese Elementary School Students (11-Year-Olds). Int. J. Environ. Res. Public Health.

[28] Takahashi T., Okade Y., Tomozoe H., Iwata Y. (2010). Introduction to Pedagogy of Physical Education.

[29] Tsunoda K., Sasaki T., Hoshino H., Minouchi Y., Miyake S. (2010). The physical fitness of male undergraduates. Jpn. Assoc. Univ. Phys. Educ. Sports.

[30] Fujii K., Akimaru T., Hanai T., Sakai T. (2006). Confirmation Regarding Secular Trend in Growth and Development of Physique and Motor Fitness in Preschool Children –An Approach from Physical Maturation Rate-. Jpn. J. Phys. Fit. Sports Med..

[31] Yamauchi T. (2017). Children Living in the Era of Obesity and Low Physical Fitness: Current Situations and Secular Changes in the Body of Children in the World. Jpn. J. Health Hum. Ecol..

[32] School Health Statistics Survey. https://www.mext.go.jp/b_menu/toukei/chousa05/hoken/gaiyou/chousa/1268648.htm.

[33] New Physical Fitness Test Implementation Guidelines. https://www.mext.go.jp/a_menu/sports/stamina/05030101/002.pdf.

[34] Takahashi D., Ito T., Ito Y., Natsume K., Noritake K., Ochi N., Sugiura H. (2024). Relationship between exercise habits and physical function in children aged 9–12 years. Nagoya J. Med. Sci..

[35] Results of School Health Statistics Survey. https://www.e-stat.go.jp/stat-search/files?page=1&toukei=00400002&tstat=000001011648.

[36] The Junior High School Curriculum Guidelines 1969. https://erid.nier.go.jp/files/COFS/s44j/index.htm.

[37] Sasaki H. (2020). Imperial Japan’s Olympics and National Physical Education: On the National Significance and Autonomy of Sports. Stud. Hist. Educ..

[38] Nakamura Y. (1992). Public Administration of National Physical Training in the War Period. Waseda J. Hum. Sci..

[39] (1977). The Junior High School Curriculum Guidelines. https://erid.nier.go.jp/files/COFS/s52j/chap2-7.htm.

[40] (1989). The Junior High School Curriculum Guidelines. https://erid.nier.go.jp/files/COFS/h01j/chap2-7.htm.

[41] (1998). The Junior High School Curriculum Guidelines. https://erid.nier.go.jp/files/COFS/h10j/chap2-7.htm.

[42] The Current State of Athletic Club Activities. https://www.mext.go.jp/sports/b_menu/shingi/013_index/shiryo/__icsFiles/afieldfile/2017/08/17/1386194_02.pdf.

[43] Pedišić Ž., Strika M., Matolić T., Sorić M., Šalaj S., Dujić I., Rakovac M., Radičević B., Podnar H., Jurakić Z.G. (2023). Physical activity of children and adolescents in Croatia: A global matrix 4.0 systematic review of its prevalence and associated personal, social, environmental, and policy factors. J. Phys. Act. Health.

[44] Bradley R., McRitchie S., Houts R., Nader P., O’Brien M., The NICHD Early Child Care Research Network (2011). Parenting and the decline of physical activity from age 9 to 15. Int. J. Behav. Nutr. Phys. Act..

[45] Araújo Bacil E.D., Mazzardo Júnior O., Rech C.R., dos Santos Legnani R.F., de Campos W. (2015). Physical activity and biological maturation: A systematic review. Rev. Paul. Pediatr..

[46] Ministry of Education, Culture, Sports, Science and Technology (MEXT) Guidelines for School Reopening and Temporary Closures in Response to COVID-19. https://www.mext.go.jp/a_menu/coronavirus/mext_00001.html..

[47] Kim H.K., Tanaka K., Inagaki A., Suzuki K., Mukouyama T., Nakamura N., Koiso T., Matsuura Y. (1993). Daily living conditions related to physical fitness and motor ability in boys 12 through 14 years of age. Jpn. Soc. Phys. Educ. Health Sport Sci..

[48] Naito H. Loss of grip strength in children. The Nikkei.

[49] Kondo M. (1995). Current status and significance of children’s play. J. Health Phys. Educ. Recreat..

[50] Hirota T., Kawano S., Shibuya T., Tsutsui T. (2011). Tracking sports-related aspirations amongst working class youth: Re-analyzing the data from previous studies. Jpn. J. Sport Sociol..

[51] Katsurada T., Mano Y. (2018). Construction and maintenance of sports facilities. J. Plan. Adm..

[52] Hirata T. (1991). Analysis of historical change of sports industry. J. Jpn. Soc. Sports Ind..

[53] Terada M., Kinoshita I. (2020). Research on local governmental restrictions for ball play in block parks. J. Jpn. Inst. Landsc. Archit..

[54] School Safety Guidelines. https://www.mext.go.jp/a_menu/kenko/anzen/1289303.htm.

[55] Tanahashi M. (2002). A Study on the Change of Japanese Children’s Play considering their Health. Aichi Shukutoku Univ. Fac. Cult. Creat. Res. Bull..

[56] Basic Plan for the Promotion of Sports. https://www.mext.go.jp/a_menu/sports/plan/06031014.htm.

[57] Rowlands A.V., Eston R.G., Ingledew D.K. (1999). Relationship between activity levels, aerobic fitness, and body fat in 8- to 10-yr-old children. J. Appl. Physiol..

[58] Handbook for Initiatives to Improve Children’s Physical Fitness. https://www.mext.go.jp/a_menu/sports/kodomo/zencyo/1321132.htm.

[59] Improvement of Children’s Physical Fitness. https://kodomo.recreation.or.jp/current/now/.

[60] Tomobe K. (2007). Secular Trends and Short-Term Fluctuations of Mean Age of Menarche and an Analysis of Peak Height Velocity in Modern Japan: A Reconsideration of Economic Darkness during the Interwar Period from the Viewpoint of Anthropometric History. Socio-Econ. Hist..

[61] Iida Y. (2018). Physical Growth and Development. Japan Soc. Athl. Train..

